# Expanding the clinical spectrum of idiopathic intracranial hypertension

**DOI:** 10.1097/WCO.0000000000001131

**Published:** 2022-11-28

**Authors:** Benson S. Chen, John O.T. Britton

**Affiliations:** a John van Geest Centre for Brain Repair and MRC Mitochondrial Biology Unit, Department of Clinical Neurosciences, University of Cambridge; b Cambridge Eye Unit, Addenbrooke's Hospital, Cambridge University Hospitals, Cambridge, United Kingdom

**Keywords:** idiopathic intracranial hypertension, MRI, raised intracranial pressure, spontaneous cerebrospinal fluid leak, temporal lobe epilepsy

## Abstract

Idiopathic intracranial hypertension (IIH) is a disorder of raised intracranial pressure (ICP). Although the majority of patients with IIH present classically with headache and papilledema, some patients may have unusual presentations or manifestations. Recent advancements in neuroimaging have facilitated the identification of other presentations associated with IIH. This review provides an overview of the expanding clinical spectrum of IIH.

**Recent findings:**

Presentations of IIH that are considered unusual include highly asymmetric or unilateral papilledema, IIH without papilledema, and IIH associated with cranial nerve involvement. These presentations likely reflect differences in the way cerebrospinal fluid (CSF) pressure is transmitted intracranially. Radiological signs of intracranial hypertension are increasingly recognized in patients with IIH and provide further insights into the effects of raised ICP on intracranial structures. Osseous changes in the skull base leading to formation of meningoceles and encephaloceles have been identified in patients with IIH, spontaneous skull base CSF leak, and drug-resistant temporal lobe epilepsy, suggesting a possible association.

**Summary:**

Clinicians should be familiar with the expanding clinical spectrum of IIH and the implications for the management of these presentations.

## INTRODUCTION

Idiopathic intracranial hypertension (IIH), also known as pseudotumor cerebri, is a disorder of raised intracranial pressure (ICP) that typically affects women of reproductive age. Although the cause of IIH is poorly understood, the condition is inextricably linked with obesity and metabolic dysregulation, evidenced by the increasing prevalence of IIH in parallel with the global obesity epidemic [1]. The classic presentation of IIH with headache and papilledema, remains substantially unchanged from Walter Dandy's original description of the condition in 1937 [2]. However, it is increasingly recognized that IIH can manifest with other symptoms and signs. This review will explore the expanding clinical spectrum of IIH, focusing on the insights they provide regarding the pathophysiology of IIH and implications for their management. 

**Box 1:**
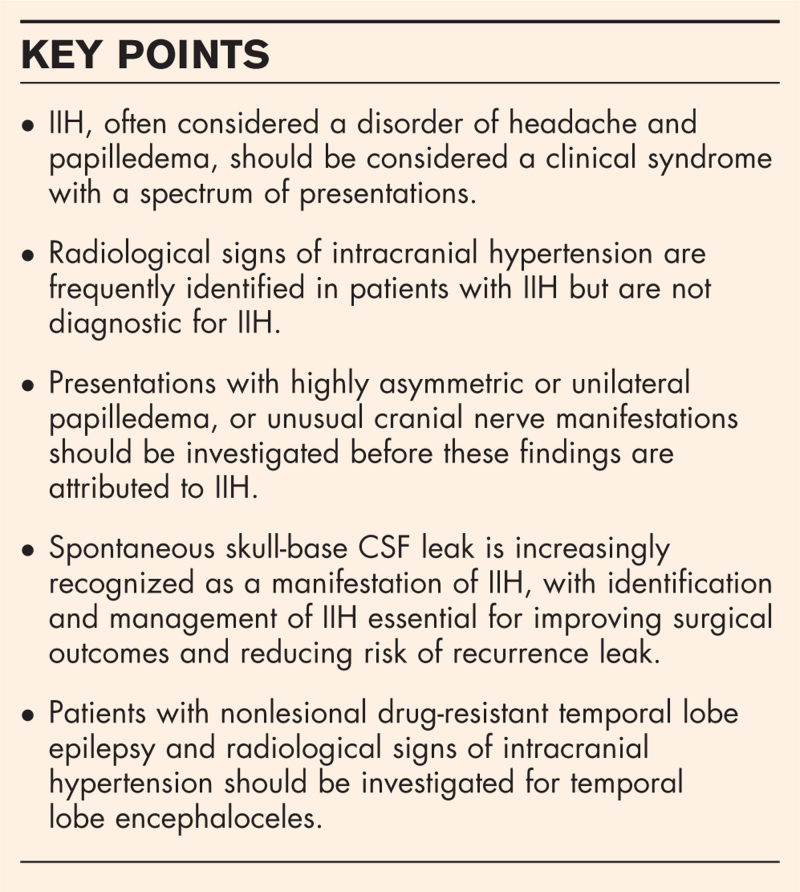
no caption available

## TYPICAL PRESENTATIONS OF IDIOPATHIC INTRACRANIAL HYPERTENSION

**FIGURE 1:**
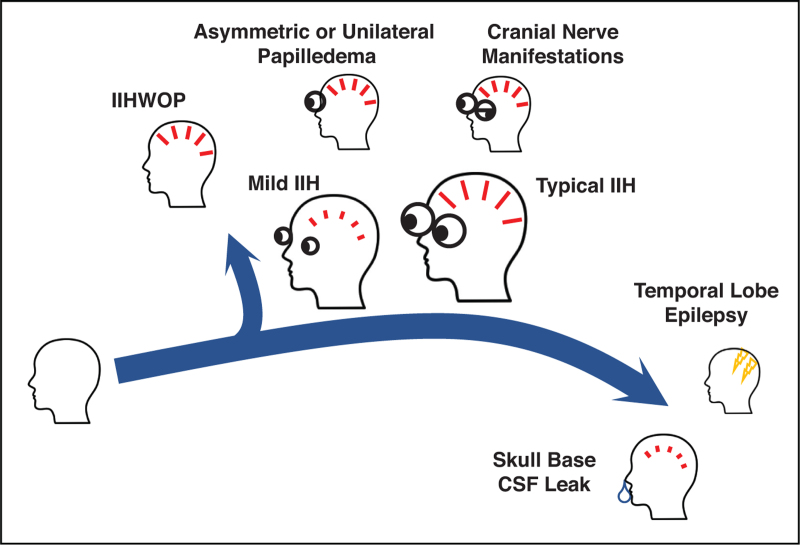
The clinical spectrum of idiopathic intracranial hypertension. Typical IIH presents with headache (indicated by lines inside the head) and papilledema (indicated by enlarged eyes), often accompanied by other symptoms of raised intracranial pressure and normal neurological examination. This is the most common presentation (roughly indicated by size of head). A subset of patients with IIH are relatively asymptomatic or have mild IIH. These patients are often incidentally found to have papilledema and, compared with patients with typical IIH, have fewer headaches, less severe papilledema, and lower cerebrospinal fluid opening pressure on lumbar puncture. Patients with IIH without papilledema (IIHWOP) have a chronic refractory headache syndrome. It is unclear if IIHWOP represents a variant of IIH where ICP is not elevated enough for papilledema to develop or a separate disorder of intracranial hypertension. IIH can also present with highly asymmetric or unilateral papilledema and other cranial nerve manifestations, including ocular motor nerve palsies, trigeminal neuropathy or neuralgia, and facial nerve dysfunction. With chronic intracranial hypertension, osseous changes can occur in the skull base predisposing to spontaneous skull base CSF leak, which presents with CSF rhinorrhea and headache. Osseous changes can also lead to formation of temporal meningoceles and encephaloceles, potentially acting as a nidus for temporal lobe epilepsy. CSF, cerebrospinal fluid; CT, computed tomography; ICP, intracranial pressure; IIH, idiopathic intracranial hypertension.

The most common and ‘classic’ presentation of IIH is headache and papilledema (Fig. 1). Over 90% of IIH patients are affected by headache, which is the key determinant of morbidity in IIH [3^▪^]. The features of headache in IIH vary substantially and frequently has a migraine phenotype. Although headaches can improve with reduction in ICP, more than half of IIH patients complain of persistent chronic headache despite normalization of ICP [3^▪^]. Papilledema is an ‘outstanding objective finding’ of IIH [2] and is the key diagnostic criterion in IIH, which also incorporates brain imaging to rule out secondary causes of raised ICP, elevated cerebrospinal fluid (CSF) opening pressure, and normal CSF constituents (Table 1) [4]. Visual symptoms that may accompany papilledema include transient visual obscurations, blurred vision, and double vision due to abducens nerve palsy [1]. Other associated symptoms of IIH include dizziness, pulsatile tinnitus, and cognitive impairment.

**Table 1:** Diagnostic criteria for idiopathic intracranial hypertension [4]

1. Required for the diagnosis of IIH
A. Papilledema
B. Normal neurologic examination except for cranial nerve abnormalities
C. Neuroimaging: Normal brain parenchyma without evidence of hydrocephalus, mass, or structural lesion and no abnormal meningeal enhancement on MRI, with and without gadolinium, for typical patients (obese women), and MRI, with and without contrast, and MRV for others; if MRI is unavailable or contraindicated, contrast-enhanced CT may be used
D. Normal CSF composition
E. Elevated lumbar puncture CSF opening pressure [≥25 cm CSF in adults and ≥28 cm CSF in children (25 cm CSF if the child is not sedated and not obese)] in a properly performed lumbar puncture
Diagnosis of IIH is definite if the patient fulfils criteria A–E. The diagnosis is considered probable if criteria A–D are met, but the measured CSF pressure is lower than specified for a definite diagnosis
2. Diagnosis of IIHWOP
In the absence of papilledema, a diagnosis of IIHWOP can be made if B–E from above are satisfied, and in addition the patient has unilateral or bilateral abducens nerve palsy.
In the absence of papilledema or sixth nerve palsy, a diagnosis of IIHWOP can be suggested but not made if B–E from above are satisfied, and in addition at least three of the following neuroimaging criteria are satisfied:
i. Empty sella
ii. Flattening of the posterior aspect of the globe
iii. Distention of the perioptic subarachnoid space with or without a tortuous optic nerve
iv. Transverse venous sinus stenosis

CSF, cerebrospinal fluid; CT, computed tomography; IIH, idiopathic intracranial hypertension; IIHWOP, idiopathic intracranial hypertension without papilledema; MRV, magnetic resonance venography.

It is recognized that some patients with IIH may be relatively asymptomatic, and papilledema may be incidentally discovered by eye care providers when the patient attends a routine eye examination or has an unrelated eye issue. In a recent study, this presentation accounted for 40.3% of all IIH patients assessed in tertiary neuro-ophthalmology clinics in one large North American city [5]. Compared with patients with the classic presentation of IIH, incidentally discovered patients had a significantly lower CSF opening pressure, were less symptomatic, had better visual function, and required less treatment.

**FIGURE 2:**
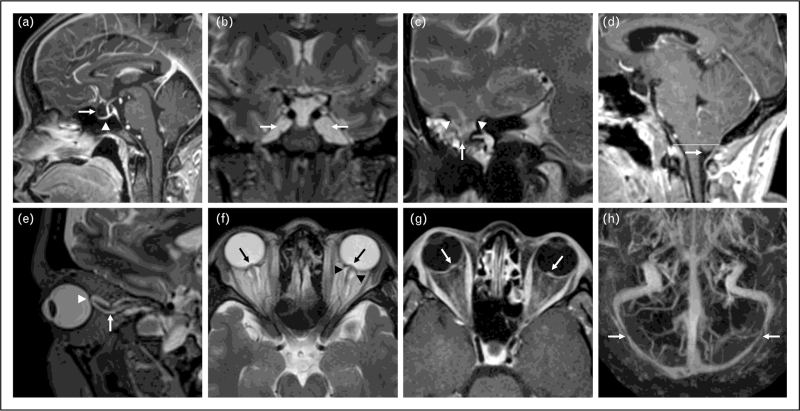
Imaging examples of radiological signs of intracranial hypertension. (a) Sagittal postcontrast T1-weighted image demonstrating an expanded, cerebrospinal fluid (CSF)-filled ‘empty’ sella turcica (arrow) and flattened appearance of the pituitary gland against the floor of the sella turcica (arrowhead). (b) Coronal T2-weighted image demonstrating abnormally enlarged Meckel caves bilaterally, filled with CSF (arrows). (c) Coronal T2-weighted image through the right temporal lobe demonstrating a meningoencephalocele. Defects in the tegmen mastoideum (margin indicated by arrowheads) transmit CSF and a portion of the right inferior temporal lobe (arrow). Fluid within adjacent mastoid air cells indicates associated CSF leak. (d) Sagittal postcontrast T1-weighted image demonstrating cerebellar tonsillar ectopia. The cerebellar tonsils, with normal rounded morphology (arrow), are in a low-lying position 5 mm below the foramen magnum (white line). (e) Oblique sagittal reconstruction of volumetric T2-weighted image in the plane of the optic nerve demonstrates posterior scleral flattening (arrowhead) and increased vertical tortuosity of the intra-orbital optic nerve sheath complex (arrow). (f) Axial T2-weighted image shows protrusion of the optic nerve head (arrows) and distention of the optic nerve sheaths by CSF (arrowheads) bilaterally. (g) Axial postcontrast T1-weighted image demonstrates enhancement of the optic nerve head bilaterally (arrows). (h) Cranio-caudal maximum intensity projection (MIP) of a postcontrast MR venogram. Arrows demonstrate smooth tapered severe stenosis of the distal transverse sinuses bilaterally.

Over the past 20 years, an increasing number of radiological signs of intracranial hypertension (RAD-IH), such as empty sella, have been recognized in IIH patients (Fig. 2) [6]. It is crucial that clinicians do not immediately associate incidentally detected RAD-IH with a presumptive diagnosis of IIH, as these findings can also be seen in those with unrelated disorders or may indicate a state of previous intracranial hypertension [7]. The vast majority of patients with incidentally detected RAD-IH do not have papilledema and, therefore, do not have IIH [8]. Proceeding to lumbar puncture or empiric treatment with acetazolamide has the potential to cause patient harm [9].

## HIGHLY ASYMMETRIC OR UNILATERAL PAPILLEDEMA

Papilledema is due to a mechanical phenomenon where increased CSF pressure in the optic nerve sheath causes axoplasmic flow stasis at the optic nerve head, leading to intraneuronal ischemia and secondary vascular changes within the optic nerves. For most patients, papilledema is bilateral and usually symmetric [1]. Highly asymmetric papilledema, defined by an inter-eye Frisén grade difference of at least 2, occurs in 3.6–10% of IIH patients [10^▪^]. Strictly unilateral papilledema in IIH is rare, occurring in 1.4% of patients in one series [11], and presents a diagnostic dilemma. These patients should undergo a detailed neuro-ophthalmic examination before the unilateral disc edema is attributed to IIH [12].

Several mechanisms for asymmetric papilledema and unilateral papilledema have been proposed, with the unifying hypothesis that differential CSF pressure is transmitted to the optic nerve head. Several studies have noted a propensity for unilateral papilledema in older patients [13,14]. With aging, the lamina cribrosa becomes stiffer and less resilient because of an increase in total collagen and a reduction in the proportion of collagen type 3 [15]. The optic nerve sheath, composed of fibrous tissue, is able to distend in the presence of increased CSF pressure. However, a stiff lamina cribrosa may act as a barrier to the transmission of CSF pressure to the optic nerve head [13–16]. Intra-ocular pressure (IOP) and the pressure gradient across the lamina cribrosa may also play a role. Several cases of unilateral papilledema have been described in IIH patients following surgical reduction of IOP for glaucoma [17,18]. However, larger series of IIH patients with asymmetric papilledema have not found a significant difference in IOP between eyes [11].

Compartmentation of perioptic CSF has also been proposed as a mechanism for asymmetric papilledema. A complex system of ‘micropartitions’ in the subarachnoid space of the optic nerve sheath are believed to interfere with the transmission of CSF between the intracranial subarachnoid space and the lamina cribrosa [19,20]. A similar hypothesis contends that a smaller bony optic canal may act as a barrier to the transmission of CSF pressure to the lamina cribrosa. One study found an association between larger optic canals on MRI and a greater severity of papilledema in patients with IIH [21]. Subsequent studies employing CT, which delineates bony anatomy more accurately than MRI, found no significant relationship between optic canal dimension and severity of papilledema [22,23].

## IDIOPATHIC INTRACRANIAL HYPERTENSION WITHOUT PAPILLEDEMA

A further enigma within the IIH spectrum is IIHWOP, a condition of chronic refractory headaches attributed to raised ICP in the absence of papilledema. It is unclear if IIHWOP is a distinct clinical syndrome or a variant of IIH, where ICP is not sufficiently elevated to develop papilledema or patients have a higher ICP threshold for developing papilledema. Although published diagnostic criteria exist (Table 1) [4], the frequency of IIHWOP among patients with chronic refractory headache varies considerably (2.5–86%) and is a reflection of the condition's diagnostic uncertainty [24^▪▪^]. There is debate regarding the optimal cut-off for CSF opening pressure in order to diagnose IIHWOP, especially because of the technical limitations of lumbar puncture and the fluctuant nature of CSF pressure [25]. Alternative noninvasive methods of measuring ICP include sonographic measurement of optic nerve sheath diameter, and optical coherence tomography to assess spontaneous retinal venous pulsations [26,27]. These options, though advancing, are not yet deployable as a replacement for conventional methods because of poor accuracy and high intra-observer and interobserver variability [28]. The identification of RAD-IH on neuroimaging have helped to improve the diagnosis of IIHWOP and distinguish the condition from chronic daily headache or migraine headache with an incidentally raised CSF opening pressure [29].

Accurate diagnosis of IIHWOP remains challenging but has implications for management. Missed diagnoses of IIHWOP may leave patients with significant headache morbidity. Although there is no consensus for the management of IIHWOP, several case series have described resolution or improvement of symptoms with appropriate control of CSF pressures [25,30,31]. Surgical CSF diversion procedures are known to be effective when managing symptoms of medically refractive IIH. However, lack of papilledema and long-standing symptoms have been identified as risk factors for treatment failure [32]. There is an opportunity to explore whether other treatments for IIH, such as weight reduction, and treatments under investigation, such glucagon-like peptide 1 receptor agonists [33], may also be applied to the management of IIHWOP.

## IDIOPATHIC INTRACRANIAL HYPERTENSION WITH UNUSUAL CRANIAL NERVE INVOLVEMENT

The diagnostic criteria for IIH requires a normal neurological examination with the exception of abducens nerve palsy, which has an established association with raised ICP. However, reports of other cranial nerve involvement have been described in the literature, most often in the setting of so-called ‘pseudotumor cerebri’ [10^▪^]. However, many of these historical cases would not satisfy the diagnostic criteria for IIH by today's measures. IIH remains a diagnosis of exclusion and the presence of other cranial nerve involvement should prompt investigation for an alternative diagnosis or explanation for the finding.

Case reports have variously described involvement of the oculomotor nerve. This most frequently presents with unilateral or bilateral pupil-sparing, extraocular muscle palsy, which resolves following ICP reduction [34–36]. An anatomical basis for this is not certain. However, a widely proposed hypothesis contends that downward displacement of the brainstem results in tractional impingement of the oculomotor nerve.

Presentations with simultaneous involvement of the oculomotor, trochlear, and abducens nerves, causing diffuse ophthalmoplegia or global ophthalmoparesis, have also been described [37–39]. These presentations are associated with markedly elevated CSF pressures (≥50 cmCSF) and are almost always because of a secondary cause [10^▪^]. Careful investigation with magnetic resonance venography for cerebral venous sinus thrombosis, and repeat lumbar puncture and antiganglioside antibody serology for Miller Fisher Syndrome may be required to exclude these differentials.

Trochlear nerve palsy has been described in case reports of mainly teenagers with IIH [10^▪^]. Some of these cases have been associated with use of medications including minocycline, topical Vitamin A, and nalidixic acid [40–42]. This underscores the importance of taking an accurate medication history in all patients presenting with an IIH syndrome. A recent systematic review found that Vitamin A derivatives, tetracycline-class antibiotics, recombinant growth hormone, and lithium are most strongly associated with drug-induced intracranial hypertension [43^▪^]. Evidence of trochlear nerve palsy resolved in all reported cases after ICP-lowering therapy and cessation of the provoking medication.

Two patients with trigeminal neuralgia or trigeminal neuropathy fulfilling the most recent diagnostic criteria for IIH have been reported [44,45]. It is unclear if trigeminal nerve involvement is directly due to the effects of raised ICP or a consequence of changes in the morphology of Meckel cave. Meckel cave undergoes morphological changes under conditions of raised ICP, becoming enlarged or indented [46]. It is hypothesized that this morphological change predisposes the trigeminal nerve to compression or distension as it traverses Meckel cave. Indeed, alterations in the morphology of Meckel cave have been identified in patients with trigeminal neuralgia (without IIH) and implicated as a cause of idiopathic trigeminal neuralgia after neurovascular contact has been excluded [47].

Several cases of unilateral facial weakness have been reported in the setting of IIH [10^▪^,^48^,^49^]. A number of mechanisms for facial nerve involvement have been proposed, including: raised ICP causing tractional forces on the extra axial facial nerve; and compression of the facial nerve as it traverses an enlarged fallopian canal. Facial diplegia and hemifacial spasm are even rarer presentations of IIH [10^▪^]. It is important to distinguish these presentations, particularly diplegia, from other diagnoses such as Guillain–Barré syndrome.

IIH presenting with lower cranial nerve involvement is rare. Isolated reports of uvula deviation, torticollis, and tongue weakness suggest an association with the vagus, spinal accessory, and hypoglossal nerves, respectively [50–52]. Traction or compression of these lower cranial nerves by raised ICP, as a hypothesis for their association with IIH, is less tenable, on account of where they emerge from the lower brainstem.

## SPONTANEOUS SKULL BASE CEREBROSPINAL FLUID LEAK

Spontaneous skull base CSF leak presenting with rhinorrhea, headache, and recurrent meningitis is an unusual presentation that is strongly associated with IIH. A striking overlap exists in the demographic, clinical, and radiological characteristics of patients with IIH and those with spontaneous CSF leaks [53^▪^]. Like IIH, rates of spontaneous CSF leak have increased in parallel with the obesity epidemic. Both conditions mainly affect women. However, patients with spontaneous CSF leak are older than IIH patients and experience fewer headaches and pulsatile tinnitus, perhaps because CSF leak acts as a ‘pressure release valve’ against raised ICP, relieving the signs and symptoms of IIH and delaying its presentation [54]. RAD-IH are frequently detected in patients with spontaneous CSF leak, in particular empty sella, meningoceles and encephaloceles [53^▪^]. Meningoceles and encephaloceles are highly prevalent in patients with spontaneous CSF leak and typically occur in the anterior cranial fossa, while in IIH, they usually involve Meckel cave or the petrous apex [55].

The pathophysiology of spontaneous CSF leak is not fully understood but likely overlaps with the mechanisms that give rise to IIH. It is hypothesized that patients with spontaneous CSF leak may have a predisposition to abnormally thin or weak skull base bones, which is further exacerbated by raised ICP. Spontaneous CSF leak frequently involves the anterior skull base, particularly the cribriform plate in the ethmoid bone and the lateral recess of the sphenoid sinus. The cribriform plate is anatomically vulnerable to raised ICP because of the presence of numerous foramina for the passage of the olfactory nerves, while pneumatization of the paranasal sinuses by raised ICP may weaken the sphenoid roof, predisposing to osseous erosion and spontaneous CSF leak [56]. Related to this is the hypothesis that IIH is a disorder of imbalanced glymphatic flow, with congestion of the glymphatic system leading to overflow of the lymphatic CSF outflow pathway [57^▪^]. This pathway includes the sheaths of the cranial nerves joining the deep cervical lymph nodes. Chronic congestion of the lymphatic CSF as it travels through the sheaths of the cranial nerves, around the olfactory bulb, is thought to contribute to erosion of the cribriform plate [57^▪^].

It is difficult to make a preoperative diagnosis of IIH in patients with spontaneous CSF leak, as ∼14% of patients have papilledema preoperatively and CSF pressure is frequently below the cut-off set out in the diagnostic criteria for IIH [58]. Postoperatively, not all patients with spontaneous CSF leak will develop papilledema or fulfil diagnostic criteria for IIH [53^▪^]. However, a substantial proportion of patients (up to 82% in one series) develop recurrence of CSF leak after repair [59], presumably because of a failure to treat the underlying elevated ICP.

Patients with bilateral transverse venous sinus stenosis on preoperative MRV have a higher risk of developing symptoms and signs of raised ICP postoperatively, suggesting that this finding may potentially alter peri-operative management [60,61]. Bilateral transverse venous sinus stenosis is a highly sensitive imaging marker of IIH found in almost all patients with IIH [6]; and venous sinus stenting (VSS) has emerged as an effective alternative treatment for a subset of patients with medically refractive IIH [62]. Some institutions have incorporated VSS into the management of patients with spontaneous CSF leak, either as a standalone procedure or as an adjunct to surgical repair as part of the treatment for elevated ICP [58,63]. There is insufficient evidence to support this practice in all patients with spontaneous CSF leak. However, it is widely accepted that assessment of ICP and definitive management of raised ICP, either conservative measures (weight loss, acetazolamide) and/or surgical measures (CSF diversion procedures), are required in order to reduce the risk of recurrent CSF leak or postoperative IIH [64^▪▪^].

## TEMPORAL LOBE EPILEPSY

Recent studies have found an association between temporal lobe encephalocele and IIH, suggesting that temporal lobe epilepsy (TLE) could be an unusual manifestation or complication of IIH. Pulsatile CSF forces secondary to raised ICP are hypothesized to lead to the development of prominent arachnoid villi, which form pockets of CSF resulting formation of spontaneous CSF fistulas and encephaloceles [65^▪^,^66^]. Temporal lobe encephaloceles are increasingly recognized as a cause of epilepsy. In a large series of 474 patients evaluated for epilepsy surgery at one center over a 5-year period, temporal lobe encephaloceles were detected in 25 (5.3%) patients. Of these patients, the temporal lobe encephalocele was considered to be the epileptogenic focus in 48%. It is hypothesized that temporal lobe encephaloceles cause mechanical irritation of the temporal lobes, and secondary changes, such as inflammation and gliosis, act as a nidus for seizures. Most temporal lobe encephaloceles are asymptomatic and incidentally discovered in patients without a history of seizures. However, in a small proportion of patients with drug-resistant temporal lobe epilepsy, temporal lobe encephaloceles related to a defect in the anterior portion of the middle cranial fossa (anteromedial and anteroinferior temporal lobe encephaloceles) appear to lateralize to the side of seizure-onset, and demonstrate high concordance with investigations including PET, scalp EEG, and seizure semiology [65^▪^,^67^,^68^].

Patients with TLE and temporal lobe encephalocele share similar demographic characteristics with IIH patients, including a female predominance and high BMI [65^▪^,^66^,^68^,^69^]. Several studies have also shown a high prevalence of RAD-IH in patients with TLE and temporal lobe encephalocele, including an expanded or empty sella, enlarged Meckel cave, optic nerve sheath distension, flattening of the posterior globe, and transverse venous sinus stenosis [65^▪^,^66^,^69^,^70^]. Other symptoms and signs of IIH, such as headache, visual disturbance, pulsatile tinnitus, and papilledema, are rarely encountered in patients with TLE and temporal lobe encephaloceles [66]. However, in support of an association with IIH, some patients with TLE and temporal lobe encephaloceles have an elevated CSF opening pressure greater than 25 cmH_2_O [7,65^▪^].

It is important for neurologists, particularly epileptologists, and those working in epilepsy multidisciplinary teams, to recognize the association between TLE and IIH. Encephaloceles may be easily overlooked on standard imaging techniques, and imaging with 3T MRI and high-resolution CT of the skull base may be required to confirm temporal lobe encephaloceles, especially in patients with nonlesional TLE who have RAD-IH on standard imaging and/or demographic characteristics suggestive of IIH [65^▪^,^67^–^69^]. Treatment for drug-resistant TLE due to temporal lobe encephalocele is primarily surgical, and most patients have a good outcome (postsurgical Engel class I) [66–68]. There is increasing evidence that restricted resection is well tolerated and effective in patients with TLE and temporal lobe encephaloceles [65^▪^].

## CONCLUSION

Although the majority of patients with IIH present classically with headache and papilledema, the disorder should be considered a syndrome with a clinical spectrum of presentations. Some patients with IIH can be relatively asymptomatic; whereas in others, papilledema may be highly asymmetric, unilateral, or not present at all. Unusual cranial nerve involvement beyond the abducens nerve has also been reported in association with IIH. Recent advancements in neuroimaging provide insights into the pathophysiological mechanisms that give rise to some of these presentations, allowing the identification of new associations including spontaneous skull base CSF leak and temporal lobe epilepsy. Clinicians should be aware of clinical spectrum of IIH and implications for the management of these presentations.

## Acknowledgements

*None.*

### Financial support and sponsorship

*B.S.C. is recipient of the Cambridge-Rutherford Memorial Scholarship awarded by the Royal Society Te Aparangi – Rutherford Foundation and the Cambridge Commonwealth, European & International Trust. No funding was received for this article.*

### Conflicts of interest

*There are no conflicts of interest.*

## REFERENCES AND RECOMMENDED READING

Papers of particular interest, published within the annual period of review, have been highlighted as:

▪ of special interest
▪▪ of outstanding interest
